# Using Natural Language Processing to Enable In-depth Analysis of Clinical Messages Posted to an Internet Mailing List: A Feasibility Study

**DOI:** 10.2196/jmir.1799

**Published:** 2011-11-23

**Authors:** Tanja Bekhuis, Marcos Kreinacke, Heiko Spallek, Mei Song, Jean A O'Donnell

**Affiliations:** ^1^Department of Biomedical InformaticsSchool of MedicineUniversity of PittsburghPittsburgh, PAUnited States; ^2^Center for Dental InformaticsDepartment of Dental Public Health, School of Dental MedicineUniversity of PittsburghPittsburgh, PAUnited States; ^3^Institute for Business TaxationLeibniz University of HanoverHanoverGermany; ^4^Office of Faculty Development and Information ManagementSchool of Dental MedicineUniversity of PittsburghPittsburgh, PAUnited States; ^5^Office of Education and CurriculumSchool of Dental MedicineUniversity of PittsburghPittsburgh, PAUnited States

**Keywords:** Dentistry, dental informatics, clinical research informatics, natural language processing, information storage and retrieval, electronic mail, information-seeking behavior

## Abstract

**Background:**

An Internet mailing list may be characterized as a virtual community of practice that serves as an information hub with easy access to expert advice and opportunities for social networking. We are interested in mining messages posted to a list for dental practitioners to identify clinical topics. Once we understand the topical domain, we can study dentists’ real information needs and the nature of their shared expertise, and can avoid delivering useless content at the point of care in future informatics applications. However, a necessary first step involves developing procedures to identify messages that are worth studying given our resources for planned, labor-intensive research.

**Objectives:**

The primary objective of this study was to develop a workflow for finding a manageable number of clinically relevant messages from a much larger corpus of messages posted to an Internet mailing list, and to demonstrate the potential usefulness of our procedures for investigators by retrieving a set of messages tailored to the research question of a qualitative research team.

**Methods:**

We mined 14,576 messages posted to an Internet mailing list from April 2008 to May 2009. The list has about 450 subscribers, mostly dentists from North America interested in clinical practice. After extensive preprocessing, we used the Natural Language Toolkit to identify clinical phrases and keywords in the messages. Two academic dentists classified collocated phrases in an iterative, consensus-based process to describe the topics discussed by dental practitioners who subscribe to the list. We then consulted with qualitative researchers regarding their research question to develop a plan for targeted retrieval. We used selected phrases and keywords as search strings to identify clinically relevant messages and delivered the messages in a reusable database.

**Results:**

About half of the subscribers (245/450, 54.4%) posted messages. Natural language processing (NLP) yielded 279,193 clinically relevant tokens or processed words (19% of all tokens). Of these, 2.02% (5634 unique tokens) represent the vocabulary for dental practitioners. Based on pointwise mutual information score and clinical relevance, 325 collocated phrases (eg, *fistula filled obturation* and *herpes zoster*) with 108 keywords (eg, *mercury*) were classified into 13 broad categories with subcategories. In the demonstration, we identified 305 relevant messages (2.1% of all messages) over 10 selected categories with instances of collocated phrases, and 299 messages (2.1%) with instances of phrases or keywords for the category *systemic disease.*

**Conclusions:**

A workflow with a sequence of machine-based steps and human classification of NLP-discovered phrases can support researchers who need to identify relevant messages in a much larger corpus. Discovered phrases and keywords are useful search strings to aid targeted retrieval. We demonstrate the potential value of our procedures for qualitative researchers by retrieving a manageable set of messages concerning systemic and oral disease.

## Introduction

In the United States, about 70% of dentists work in relative isolation as solo practitioners or in small groups [1]. Unfortunately, independent practitioners cannot afford to subscribe to all of the information resources readily available to dental faculty, academic researchers, and clinicians in large organizations. For example, the University of Pittsburgh’s Health Sciences Library System [2] also serves UPMC, a global health enterprise. Dentists affiliated with either of these organizations have access to more than 3800 books on general dentistry, endodontics, pediatrics, periodontics, restoration, and special care; 15 full-text electronic books on dentistry, including important core resources; and more than 75 dentistry journals, most of which are available electronically.

In contrast, independent practitioners typically meet their information needs by relying on colleagues, discussion lists, news outlets, and a few professional journals to which they subscribe [3]. Even though most dentists in the United States have access to the American Dental Association’s library by virtue of their membership, retrieval of more than the occasional full text is expensive. For example, if a member finds information in PubMed [4] not freely available in PubMed Central or an open source journal, the fee for retrieval and delivery by the library is US $7 to US $15 per article, and US $15 for one or two books, with possible late charges [5]. Fees are higher for nonmembers.

Thus, we conclude that the full panoply of important resources is inaccessible to most dentists when questions arise regarding best practice, especially at the point of care when readily available information is needed. This fact combined with dentists’ preference for first consulting peers means that online communities are potentially valuable sources of information [6-8]. Such communities could be used in the future as conduits for delivery of evidence-based information, such as updated guidelines for clinical care. As for delivery of information at the point of care, this urgent need demands informatics solutions and is the focus of a US federally funded project led by Dr. Heiko Spallek [9].

### Communities of Practice

An online or e-community is sometimes characterized as a virtual community of practice (CoP*)* [10] because members are geographically isolated yet connected socially via the Internet. A virtual CoP can serve as an information hub with easy access to expert advice and opportunities for social networking (eg, see [11]). The rationale for considering the opinions of peers expressed online is similar in spirit to the way in which research is initiated by practitioner-investigators in practice-based research networks [1,12,13]. In both cases, the value of clinical experience is recognized.

For our purposes, we are interested in knowing which clinical topics are discussed by dentists in a CoP. To do this, we mine their asynchronous messages posted to an enduring and active online discussion list. Once we understand the topics covered in the corpus of messages, we can study dentists’ real information needs and the nature of their shared expertise, and can avoid delivering useless content to the community or at the point of care in future informatics applications.

### Assisting Qualitative Researchers

To plan a labor-intensive study of information needs with its in-depth content analyses of clinical topics and emergent themes, one must carefully consider available human resources. For example, we have two academic dental researchers who can devote just a few days to coding and interpreting thematic content of messages with guidance from an experienced qualitative researcher. The problem then is how to assist qualitative researchers by finding a manageable number of clinically relevant messages that are worth studying given available resources.

If we know the typical length of messages, the time it takes to code a message regarding clinical topics and themes, and the number of hours researchers can devote to the content analyses, we can estimate the sample size (n) that will ensure the feasibility of the planned content analyses. Here, the corpus consisted of thousands of messages posted to an Internet mailing list for practicing dental professionals, primarily general dentists. We assumed that two academic dentists and one qualitative researcher could manage a few hundred messages.

In general, we considered three options for drawing the sample: (1) randomly sample n messages from the corpus, (2) restrict the interval of time in which n messages occur and select all messages within that interval, and (3) use natural language processing (NLP) to identify clinical topics and, depending on the research question, retrieve n messages with useful content.

The advantage of the first two options is that they are well known and easy to implement. A major disadvantage is that the selected messages may be irrelevant to the researchers’ interests, especially given the informal quality of messages posted online. In the present study, the purpose of the mailing list from which the corpus originated is to offer dentists a place to discuss their clinical concerns. However, many of the messages were off topic. For example, dentists chatted about the big football game, the trip to Europe, the swimsuit issue of *Sports Illustrated*, Michael Jackson’s death, and aging parents. Although the third option is novel and more time consuming than the first two, it is in keeping with the notion that the nature of the corpus needs to be understood *before* messages are selected. This is because inferences depend on the selected units of analysis such as blocks of text [14]. Thus, the third option ensures the feasibility and probably the quality of content analyses by identifying a manageable number of messages relevant to the research question.

In this paper, we present a workflow for identifying and retrieving a manageable subset of relevant messages from a much larger corpus. It involves a sequence of machine-based steps along with human classification of clinical phrases discovered with NLP. We also demonstrate the value of this approach for enabling study of text messages by qualitative researchers. As an example, we describe the strategy we used to retrieve messages for a study underway that involves in-depth content analyses.

A preliminary version of this paper was presented at the 2010 Annual Symposium of the American Medical Informatics Association [15].

## Methods

We mined the clinical content of 14,576 electronic messages posted to a fee-based discussion list during an approximate 1-year study period from April 18, 2008 to May 28, 2009. The subscribers to this global list are dental practitioners, mostly dentists from North America interested in clinical practice.

### Deidentification

Because the origin of our corpus of messages is a private Internet mailing list, we took care to preserve confidentiality even though (1) the University of Pittsburgh Institutional Review Board approved this study as being exempt (PRO08040313), (2) the owner of the list deleted identifying information from the message headers before sharing content, (3) messages are regularly delivered to about 450 subscribers and then saved in a searchable archive, and (4) anyone interested in clinical dental care can subscribe. The number of subscribers and the ease with which one can subscribe suggest that this mailing list has a public aspect. Nevertheless, we went through several rounds of deidentification for two reasons: (1) to ensure confidentiality [16] for future data sharing, and (2) to optimize NLP by stripping out irrelevant information. We also used Google to confirm that excerpts presented in this paper are not easily retrievable.

During NLP (see below), we deleted stopwords (eg, articles and prepositions) to optimize discovery of topical content. Surprisingly, deletion of stopwords may help preserve anonymity. This idea is based on knowing that forensic researchers use stylistic properties of messages, including number and distribution of function or stopwords, to identify authors of email [17]. We also deleted any remaining names and places by using lists and a gazetteer, respectively, available in the Natural Language Toolkit (NLTK) [18].

### Preprocessing

Mining email is challenging because of the nature of the messages [19]. For example, email can be ill formed linguistically with spelling and grammatical errors, and style can be idiosyncratic [17]. Typically, email is particularly noisy in that much of the data are irrelevant to the research question. For these reasons, processing messages is essential before clinical topics can be discovered.

Initially, we extracted the body of each message and deleted threaded responses, which is appropriate given our interest in discovering clinical topics rather than analyzing discourse. To clean the data further, we analyzed message patterns to identify recurring sources of noise (ie, data that obscure message content and meaningful frequencies in the original texts)*.* Consequently, we deleted forwarded and quoted messages; embedded visual data such as x-ray images and photographs; virus- or spam-free notices; Microsoft Outlook notices; advertisements and footers; and signature lines. The latter often include self-promotional text.

### Natural Language Processing

We used the open source NLTK version 2.0 with Python version 2.6 (Python Software Foundation, Wolfeboro Falls, NH, USA) to analyze preprocessed text. For readers new to NLP, the textbook *Natural Language Processing with Python* is a useful resource [18]. At the NLTK website [20], one can access the textbook, as well as download the programming language Python, optional packages, and the NLTK modules for NLP and text analytics.

Note that in this section we italicize terms that may be unfamiliar to readers.

We sorted and concatenated the messages by date to enable tracking discussion of topics over time. We also converted to lower case and selected *alphabetic token*s (processed words or strings of letter characters) with length >3 characters. We deleted English *stopwords* (short function words such as “a” and “the”), as well as names and places. We explored the usefulness of the obtained *vocabulary* (set of unique tokens), as well as *bigrams* and *trigrams* (pairs and triples of contiguous processed words) by examining the 100 and 300 most frequent tokens and *n-grams* (bigrams and trigrams)*.* However, these were deemed clinically uninteresting.

To find clinical *content-bearing tokens* (substantive words such as apolipoprotein and stenosis) and phrases, we selected tokens with length >5 and frequency >7, and then derived n-grams. The rationale for this filter is similar to one presented in the NLTK text [18] where the goal is to find words and phrases that characterize a *genre*. Here the genre is *email with a clinical focus written by dental practitioners*. We also created *collocated n* *-* *grams*. *Collocations* are contiguous tokens that occur together more often than one would expect if the tokens were probabilistically independent. We selected the top 600 collocated bigrams and trigrams (300 for each type) by computing the pointwise mutual information measure for each n-gram and then sorting.

We informally confirmed that collocations derived from the content-bearing tokens were likely to retrieve useful messages by constructing *concordances* for selected tokens. A *concordance* is a set of retrieved lines with windows of text around a token or target word. The windows allow one to explore the contexts in which a target word occurs in the corpus. To build a concordance using the NLTK [20], one specifies the window size or number of characters per line, as well as the number of lines to display. For example, we examined the concordance for *lesion* to preview message content. Here are two samples from its concordance:

...[t]his is almost always seen in younger patients. I’m betting this lesion is of endodontic origin. Tough case to diagnose with certainty...

...they’re looking for cancer. They will NOT understand that if a lesion looks like cancer the Brush Test is not indicated. If you see a...

### Classification of Phrases and Selection of Keywords

Although most of the collocations seem to characterize dentists’ clinical language, some are irrelevant. For example, here is a sample of collocations with irrelevant trigrams in italics: molecular bacterial antigens, *committing stating profitable*, *perspective agreement lobbyists*, methotrexate causative factor, inhibits demineralization enamel, *driving cadillac attack,* mutans streptococci presence.

Thus, two academic dentists (HS, JO) selected a subset of relevant collocated phrases, including bigrams and trigrams that could be used as search strings to retrieve messages with clinical content. Note that some n-grams overlap. By retaining overlapping n-grams, if they exist, we ensure a broader search than if we use just trigrams. (Most overlapping n-grams point to the same messages, but not always.) An example of an overlapping pair of n-grams is *prescribed amoxicillin hydrocodone* and *amoxicillin hydrocodone*.

The dentists also classified the phrases they selected by sorting them into broad categories with subcategories; this is considered an inductive approach to classification. Then they labeled the categories and subcategories. The process for both selection and classification was an iterative one involving discussion to reach consensus. The emergent classification scheme describes the clinical topics of concern to the dental practitioners who posted to the online mailing list. It likely will be useful to the qualitative researchers when they code messages for later content analyses [21].

After the phrases were classified, we identified embedded keywords (unigrams) to ensure that retrieval could be even broader, if desired. We defined a keyword as one that occurs at least twice in the full set of collocations. Each variant or closely related word counts as an occurrence. For example, *plaque* and *plaques,* as well as *atherosclerosis* and *atherosclerotic*, are variants; *cardiac* and *myocardial* are closely related. All six italicized examples can be used as search strings to find messages.

### Finding Relevant Messages: A Demonstration

**Figure 1:**
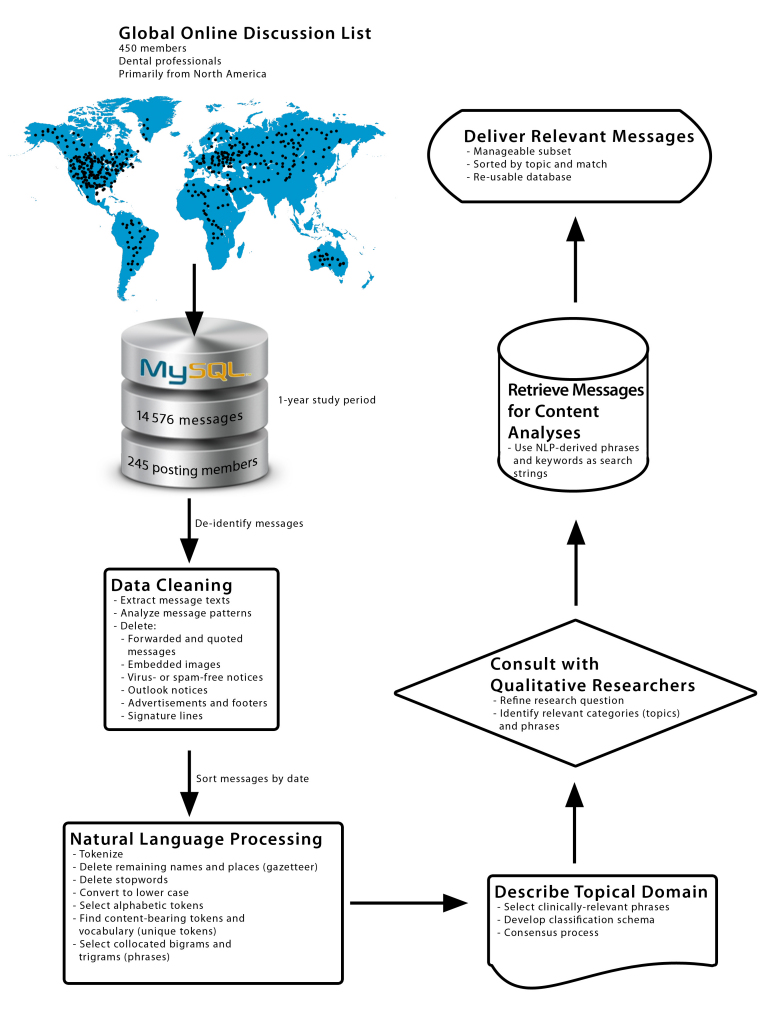
Workflow for finding clinically relevant messages posted to an Internet mailing list.

To demonstrate how the workflow presented in this paper can help researchers (see Figure 1), consider the following scenario. In our research center, a qualitative study investigating the information needs of dentists regarding the relationship between systemic disease and oral health is underway. Given this focus, two researchers independently selected some of the NLP-discovered phrases that we had identified and classified in this study. They reached consensus by discussion to determine the final list of phrases. Thus, they found a subset of phrases with embedded keywords in a subset of categories. We used the selected phrases and keywords as search strings to find messages relevant to their research question.

Because the content-bearing phrases were discovered in a merged file that had been considerably processed, a question arose as to what should be the maximum number of allowable characters between words in a phrase when searching cleaned messages not yet processed with NLP. In an informal assessment, we used 20 phrases across categories as search strings and found that the number of characters between any two words in a phrase ranged from 1 to 78. As a conservative estimate, we therefore chose to limit the interval to at most 100 characters. The aptness of this choice was borne out by the results (see below). Briefly, we carried out the following steps to retrieve and organize messages:

1. Create search strings based on collocations by first splitting phrases into words. Then for each phrase, recombine the words in any order with at most 100 characters between words. (We ignored order because words in discovered phrases were sometimes reordered in the messages, eg, *mutans streptococci* versus *streptococci mutans*.)
2. Use each keyword as a search string. If a keyword appears adjacent to another keyword in a phrase, preserve the order and search for the concatenated string.
3. Match the search strings to cleaned message texts; retrieve messages with at least one matching string.
4. Sort messages into folders (directories) per category, as well as into folders by type of match (phrase or keyword). (For example, messages with at least one phrase from the category *systemic disease* were sorted into a folder for that category, as well as a folder for all messages with instances of clinically relevant phrases. Similarly, messages with at least one keyword match were sorted into corresponding folders.)
5. Deliver deduplicated messages in folders to the researchers. (This sorting helps them find the messages they want to analyze. Further, filenames include the date when the message was posted plus a unique database identifier, which allows tracking of change in topical discussion over time, as well as retrieval of particular messages.)

For illustration purposes, consider the excerpted messages below that can be retrieved by using the following as search strings: (1) *fistula filled obturation* [trigram], (2) *herpes zoster* [bigram], and (3) *mercury* [keyword]*.* Remember that a maximum of 100 characters is allowed between the italicized words:

1. *...If you have a tooth with an actively draining* fistula *(pus* filled *canal), do you do one visit endo if you can get a dry canal before* obturation*? Or do you medicate for some time period and fill at a later date?...*
2. *...patient [with] recurrent ulcers on his palate [that] follow the distribution of the greater palatine nerve... I suspect* herpes zoster.* Most of the time I’ve seen this it’s been unilateral, but in his case it’s always bilateral. What other Dxs [diagnoses] should I be considering...*
3. *…Am I missing the point or is the issue (the real issue) with* mercury *not whether it causes systemic disease but rather the environmental issue of* mercury *in the food chain? We all (in the UK) have to have amalgam separators now but we know they’re not foolproof...*

## Results

### Subscriber Participation

**Figure 2:**
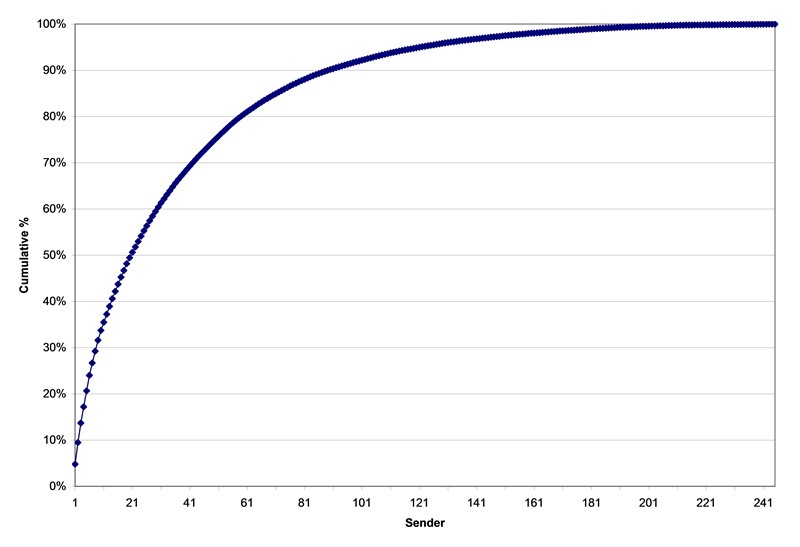
Cumulative distribution of messages posted by dental practitioners to an online discussion list.

Just over half of the subscribers (245, or 54.4%) of the approximate total number of subscribers (N = 450) posted 14,576 messages. Of these, 21 subscribers (5% of the list) posted 7288 (50%) of the messages; 29 subscribers (6% of the list) posted 3644 (25%) of the messages; and 195 subscribers (43.3% of the list) posted the remaining 3644 (25%) of the messages (see Figure 2). Thus, 205 subscribers (45.6%) were passive (ie, they received messages but did not otherwise contribute to the message traffic during the study interval). Note that the total number of subscribers is approximate because the list size varies somewhat over time.

### Natural Language Processing

The concatenated file of cleaned messages yielded 1,468,244 tokens. Initial NLP (selecting alphabetic tokens with length >3, deleting names and places, etc) reduced the number of tokens to 533,251 (36.32%).

Filtering to find clinical content-bearing tokens yielded 279,193 tokens (19.02%). For our purposes, the unique tokens in the content-bearing set (5634, or 2.02% of the content-bearing tokens) represent the dental practitioners’ vocabulary. We obtained 208,026 bigrams and 252,931 trigrams, and derived collocations. For illustration purposes, we present a handful of collocated bigrams and trigrams: *osteoclastic activity*, *painful sequestrum*, and *intravenous bisphosphonates* (bigrams); *glucose homeostasis inflammation*, *irreversible pulpitis apical*, and *supragingival scaling prophylaxis* (trigrams).

### Classification of Phrases and Selection of Keywords

The classification of phrases resulted in 13 broad categories with subcategories. Table 1 presents the categories and distribution of collocated phrases and embedded keywords. The entire classification including categories and subcategories, 325 collocated phrases, and 108 embedded keywords is presented in Multimedia Appendix 1.

**Table 1:** Distribution of collocated phrases and keywords by category

Category	n of collocated phrases^a^ (% of phrases)^b^	n of keywords^c^ (% of keywords)^d^
Systemic disease	49 (15)	21 (15)
Endodontics	18 (6)	9 (6)
Orthodontics	8 (3)	3 (2)
Periodontics	12 (4)	6 (4)
Restorative dentistry	66 (20)	20 (14)
Oral and maxillofacial surgery	26 (8)	18 (13)
Other oral diseases	7 (2)	4 (3)
Radiology	7 (2)	4 (3)
Causative agent	20 (6)	9 (6)
Medication	36 (11)	19 (13)
Materials	44 (14)	17 (12)
Basic sciences	13 ( 4)	6 (4)
Research	19 (6)	7 (5)
Total	325	143

^a^ Collocated phrases are bigrams and trigrams; selection based on pointwise mutual information score and clinical relevance.

^b^ Percentage of phrases computed relative to the total number of phrases and rounded.

^c^ Some keywords occur in more than one category. Thus, the total number of instances is greater than the number of unique keywords.

^d^ Percentage of keywords computed relative to the total number of instances of keywords and rounded.

### Finding Relevant Messages: A Demonstration

Two academic dentists conducting a qualitative study selected a subset of phrases (n_p_ = 144) with embedded keywords (n_kw_ = 95) in 10 of 13 categories potentially related to their research question.

Over k selected categories (k = 1 ... 10) and after deduplication, we retrieved 305 messages (range n_k_, 1–119 messages) with 520 instances of matching phrases; 948 messages (range n_k_, 12–343) with 1411 instances of matching keywords; and 996 messages (range n_k_, 12–363) with 1931 instances of matching phrases or keywords (see Table 2). The number of characters between words in a phrase ranged from 0 to 75, after deleting white spaces and punctuation.

**Table 2:** Number of messages with phrases or keywords retrieved for content analyses by selected category

Selected category^a^	n of messages^b^ (n of phrases)^c^	n of messages (n of keywords)	n of messages (n of phrases or keywords)
Systemic disease	119 (164)	284 (384)	299 (548)
Periodontics	14 (27)	51 (51)	54 (78)
Oral and maxillofacial surgery	36 (40)	106 (113)	106 (153)
Other oral diseases	17 (24)	44 (56)	48 (80)
Radiology	1 (1)	12 (12)	12 (13)
Causative agent	55 (78)	79 (95)	102 (173)
Medication	70 (110)	343 (377)	363 (487)
Materials	4 (4)	44 (50)	44 (54)
Basic sciences	8 (12)	157 (164)	160 (176)
Research	40 (60)	89 (109)	100 (169)
Total	305 (520)	948 (1411)	996 (1931)

^a^ Categories selected from the full set by qualitative researchers.

^b^ Number of messages after deduplication.

^c^ Collocated phrases are bigrams and trigrams; selection based on pointwise mutual information score and clinical relevance.

To interpret Table 2, consider the row for the category *medication*. In this category, we retrieved 70 messages with 110 matches for collocated phrases, such as *intravenous bisphosphonates* from the subcategory *cancer drugs* (see Multimedia Appendix 1). We also retrieved 343 messages with 377 matches for keywords, such as *proinflammatory* from the subcategory *immune system*. Finally, we retrieved 363 messages with 487 matches for phrases or keywords selected by the dentists in the category *medication*.

## Discussion

### Summary of Main Findings

A workflow with a sequence of machine-based steps and human classification of NLP-discovered phrases can support researchers who need to identify relevant messages in a much larger corpus. NLP-discovered phrases and keywords are useful as search strings to aid targeted retrieval. We demonstrate the feasibility of our procedures for qualitative researchers by retrieving a manageable set of messages concerning systemic and oral disease.

### Surveys Versus Textual Analysis

The reader might wonder, “Why bother with developing this workflow to support qualitative researchers? Why not survey the members of the virtual CoP and ask them outright about their information needs?”

In the research literature, studies of information needs and barriers typically focus on clinicians and primary or ambulatory care settings. Of these, just a few studies consider dentists [3,8,22,23]. So far, most of what we know is derived from survey questionnaires with items in a forced-choice format. The use of other methods is less common (eg, see [24]), even though relevant methods exist in commerce and public health. For example, marketing analysts of social media use text analytics to understand customer sentiment in unstructured text (see [25] for an accessible introduction), and researchers in infodemiology are developing mixed methods for monitoring content posted to the Internet [26,27].

Aside from the cost of developing sound surveys with appropriate sampling plans, a serious limitation is that respondents may not accurately remember the nature of their needs for evidence-based clinical information or the contexts in which needs arise. Interesting alternatives to surveys include analysis of cultural artifacts (eg, texts, images, or videos), face-to-face interviews, and field observation [28].

The investigators on our team whose project we used to demonstrate the feasibility of our procedures elected textual analysis as a way to understand clinical messages. For them, the corpus of messages posted by practicing dentists regarding specific patients or conditions is a rich data source. Appealing aspects of the corpus include the following: (1) information needs are contextually embedded, (2) messages are written in the “natural language” of dentists, and (3) discoverable clinical topics may not be what we would find with a questionnaire.

Another reason for our team’s interest in textual analysis is that findings from a qualitative study can be compared with those from our own surveys (eg, see [23]), as well as from studies conducted by other teams. This will allow future assessment of threats to validity associated with method, and whether information derived from different sources is complementary.

### Subscriber Participation

The very skewed distribution of subscriber participation in this study is quite similar to findings reported by Falkman et al [10], as well as Nonnecke and Preece [29]. Using the language of Wenger et al [30], Falkman and colleagues describe three groups according to their level of participation: a core group of leaders, an active group who regularly participate, and a disproportionately large group of members on the periphery. Presumably, the 5% of dental practitioners in this study who posted about half of the messages to the online discussion list were the leaders of their virtual CoP. The middle group varied considerably in their degree of participation, but they did contribute to the message traffic. Arguably, the 46% of the subscribing practitioners who never posted messages during the study interval were the peripheral group of “lurkers” or bystanders.

Interestingly, lurking on the periphery does not imply that the online community has little to offer this group. Even though passive, lurkers can still learn from core and active members who serve as information providers [10,11,30]. In fact, peripheral participation may be essential for the viability of a CoP [31] because lurking, even with its negative connotations, is “a form of participation that is both acceptable and beneficial to online groups” (p. 6, [29]).

The qualitative researchers in our group believe that clinical topics initiated and discussed by leaders and active members are probably of interest to members on the periphery. For one, they assume passive members read at least some of the messages delivered to them. They further assume that disaffected members will unsubscribe. To the extent that they are wrong, the topical domain that we have discovered may reflect the interests of core and active members rather than the entire CoP. Nevertheless, it seems reasonable to study this online dental community, as the pattern of participation is typical of other communities of practice and electronic discussion lists.

### Natural Language Processing

To cope with the noisy and informal nature of email, we heavily processed the messages. In so doing, we may have inadvertently overlooked important content-bearing phrases by deriving collocations from a much-reduced set of tokens. Nevertheless, collocations are much more informative than frequent phrases [18]. The latter are usually uninteresting, at least in this context, and seem to derive from ordinary language, repeated self-promotion, and banner advertisements. Despite our best efforts, we were unable to delete all of the text-based noise.

Many of the messages include excerpts from news items, magazine articles, or research articles. These excerpts seem to have a disproportionate number of clinical phrases relative to message content written by subscribers. (Chew and Eysenbach [26] identified a similar problem when analyzing the content of posts to Twitter (“tweets”; see [32]) during the 2009 H1N1 pandemic. They cautioned that key phrases in spam and popular news might affect retrieval of tweets and activity over time.) Because we were unable to identify automatically all of the imported content, we analyzed the entire message after preprocessing. However, one could argue that members, especially leaders, bring in relevant text and that mining messages with imported text still leads to a reasonable set of NLP-derived phrases.

### Finding Relevant Messages: A Demonstration

In this study, we demonstrate the potential usefulness of our procedures by retrieving a manageable set of relevant messages for qualitative researchers. Their research entails exploring dentists’ knowledge of the relationship between systemic and oral disease expressed in messages. To understand how they can work with messages sorted by category and type of match, consider the following scenario.

Assume the researchers can handle about 300 messages for labor-intensive content analyses. They could design a broad or focused study by considering the number and type of match in each category. For example, for a broad study, they could analyze the 305 messages with clinical content-bearing phrases that we retrieved for the categories they had selected (see Table 2). For a more focused study, they could elect to work with messages from just the first category, *systemic disease,* which has 299 messages with 548 instances of phrases or keywords. Alternatively, they could select messages in some other combination of categories and type of match with the constraint that the total number of messages to analyze is about 300. If they decide to add a clinician to the team or devote more time to the project, they could analyze a larger set of messages.

By sorting the messages we retrieved into the categories selected a priori by the qualitative researchers, we were able to create a useful database that encourages flexible investigation.

### Limitations

A major limitation of this study is that we used a single source to mine electronic messages. It is possible that the NLP-discovered phrases and their subsequent classification will not generalize to other communities. In other words, the topical domain that we discovered may not describe the clinical interests of other practitioners, such as dentists who prefer to remain offline. Even if our version of the topical domain is useful, we still need to assess whether and how it changes over time. Additionally, other methods such as latent semantic analysis, sometimes referred to as latent semantic indexing [33-35], could yield a different set of topics. Finally, although we took care to reach consensus when classifying phrases, other dental researchers could have seen a different structure. Nevertheless, the limitations of any feasibility study are offset by the potential for usefulness and discovery. We believe the limitations of this study can be addressed in the future with formal evaluations that compare methods and communities.

### Future Research

Each step in the workflow presents opportunities for further research. Nevertheless, once the system we are developing becomes reasonably efficient and robust, a cost-benefit analysis will be appropriate. For example, we could compare the labor involved and quality of retrieval for a simple random sample of messages with ad hoc keyword searches as a baseline versus our system.

Other methods to identify clinically relevant messages, such as summarization and clustering of similar summaries [19,36,37], or use of an ontology to enable retrieval (eg, see [38]) could be worthwhile. Also, discourse analysis [18] of the threaded messages could help us better understand how clinicians respond to the information needs of their peers, and whether the shared information is in keeping with the best evidence in published guidelines.

Ultimately, this program of research will help us improve knowledge transfer of useful information for the legions of dentists who practice in relative isolation.

## Multimedia Appendix 1

Classification of dental phrases with keywords. A table of keywords by category is displayed at the end of the classification.

## Abbreviations

CoP: community of practice
NLP: natural language processing
NLTK: Natural Language Toolkit
