# Supplementary material for: Using Natural Language Processing to Enable In-depth Analysis of Clinical Messages Posted to an Internet Mailing List: A Feasibility Study
Source: J Med Internet Res. 2011 Nov 23;13(4):e98. doi: 10.2196/jmir.1799 (PMC3236668; doi:10.2196/jmir.1799)
Supplement: Supplementary file 1 [file jmir_v13i4e98_app1.pdf]

Multimedia Appendix 1. Classification of dental phrases with keywords in blue small caps. Table of keywords by category displayed at the end of the classification.

1. Systemic Disease

1.1 cardiac system

lowered PLAQUE interface
sticky PLAQUES arrived
ATHEROSCLEROTIC plaques
ARTERIAL plaques

accelerates ATHEROSCLEROSIS apolipoprotein
accelerates atherosclerosis
atherosclerosis APOLIPOPROTEIN deficient
apolipoprotein deficient

TREADMILL result hematoma
dropped treadmill result

atrial fibrillation
MYOCARDIAL infarction
remain placement catheter
sudden CARDIAC arrest
cardiac arrest
aortic valves
vessels stenosis
coronal vessels STENOSIS
adverse events crestor
ENDOTHELIAL DYSFUNCTION
interval PULSES
PERIPHERAL arterial
attacks strokes
marfan SYNDROME

1.2 nervous system

NEUROTOXIC EFFECTS nervous
EPISODIC migraine
EPISODE PORPHYRIA
trigger episode porphyria
peripheral NEUROPATHY
ALZHEIMER alzheimer alzheimer
hypnotic trance
NEURONS firing

1.3 infectious disease

community acquired PNEUMONIA
CHLAMYDIA mycoplasma
chlamydia PNEUMONIAE
SHINGLES acyclovir
herpes ZOSTER
zoster shingles
hematogenous spread

1.4 general care

incident triggered history
terminal RESUSCITATE placed
terminal resuscitate

1.5 orthopedic

tendon rupture
rheumatoid ARTHRITIS
artificial joints

1.6 uro-genital system

genital urinary
estrogen deficiency
metabolic syndrome
preterm births

2. Endodontics

2.1 diagnosis

irreversible PULPITIS apical
pulpitis MAXILLARY radiographically
orifices PULPAL
possibilities pulpal exposure
LESION resorption progressed

2.2 treatment

filled OBTURATION MEDICATE
obturation medicate period
fistula filled obturation
obturation medicate
pulpal EXPOSURE entering
exposure entering latter

draining fistula filled
draining FISTULA
actively draining fistula
actively draining
irreversible pulpitis

2.3 cosmetic

retreat SURGICALLY BLEACH
surgically bleach internally

3. Orthodontics

3.1 retainer

retainer answers ORTHODONTISTS
retainers scares proper
removable nightly RETAINER
nightly retainer answers

3.2 progressive

progressive ORTHODONTICS
hearing progressive orthodontics

3.3 development

MANDIBULAR erupted intact
premolars addition substitution

4. Periodontics

4.1 prevention

maintaining PERIODONTALLY discussed
terrible maintaining periodontally
hygienist CALCULUS tartar
calculus tartar difficult
calculus tartar
supragingival scaling prophylaxis

4.2 diagnosis
pericoronitis PERICORONITIS
fracture periodontally stable

4.3 ultrasound
intensity pulsed ULTRASOUND
PULSED ultrasound

4.4 etiology
OSTEOCLAST formation
OSTEOCLASTIC activity

5. Restorative Dentistry
5.1 diagnosis
CORONAL remnants deciduous
coronal remnants
NONRESTORABLE possibilities thought
nonrestorable possibilities

5.2 instruments
torque wrench
turbine HANDPIECES

5.3 fabrication
fabrication MATRIX slightly
veneer fabrication matrix
sandblasting zirconia
inlays onlays
prepped trimmed

5.4 occlusion
occlusion LOOSENED severely
loosened severely period
loosened severely
centric longer touching
prepping cuspids
vertical dimension
LATERAL excursions
PARAFUNCTIONAL forces

5.5 esthetic
academy comprehensive esthetics
refresh SHADES
refresh shades replaced

5.6 function
FUNCTIONS speaking swallowing

5.7 temporary treatment

TEMPORARILY restore CARIOUS
interim TEMPORARY maintainer

5.8 relines

permanent chairside RELINES
chairside relines therefore
chairside relines

5.9 splint

provisional SPLINTS
distally besides SPLINTING

5.10 mineralization

demineralization enamel enhances
recovery demineralized enamel
recovery demineralized
readily demineralized enamel
readily DEMINERALIZED
demineralized enamel CARIOGENIC
enamel cycles DEMINERALIZATION
cycles demineralization
inhibits demineralization enamel
saliva inhibits demineralization
inhibits demineralization

demineralization REMINERALIZATION continue
cycles demineralization remineralization
demineralization remineralization
remineralization recovery demineralized
enhances remineralization recovery
enamel enhances remineralization
enhances remineralization

CALCIUM phosphate MINERALIZED
phosphate mineralized enamel
phosphate mineralized
MINERAL density
calcium phosphate

5.11 buffering

neutral BUFFERING capacity
saliva neutral buffering
neutral buffering
buffering capacity present
buffering capacity
SALIVA calcium phosphate

5.12 other

EMERGENCE profiles
emergence profile
scares proper MAXILLARY
accurately damage framework
MAXILLA MANDIBLE predictable
DISINFECTING impressions
fluorescence measurements

6. Oral and Maxillofacial Surgery
6.1 diagnosis

painful SEQUESTRUM lingual
painful sequestrum
presented painful sequestrum
result hematoma partner
HEMATOMA partner painful
hematoma partner
septic ARTHRITIS OSTEOMYELITIS
osteomyelitis ANAEROBIC
osteonecrosis secondary BISPHOSPHONATE
OSTEONECROSIS secondary
depressed sockets
remnants deciduous
linear lesion triangular
LESION triangular shaped
triangular shaped ULCERS
LATERALS canines
ASYMPTOMATIC BILATERAL opposite
asymptomatic bilateral

6.2 other

minimally INVASIVE
tonsils TONSILS
SALIVARY glands
thinks retreat SURGICALLY
extracting MANDIBULAR erupted
lancet INFECT author
lancet infect
socket inferior ALVEOLAR

7. Other Oral Diseases
7.1 oral cancer

LICHEN planus biopsy
lichen planus
marrow transplant

7.2 skin / mucosa

HERPES simplex causes
herpes simplex
recurrent APHTHOUS
aphthous ULCERS

8. Radiology

phosphor plates
optical SCANNING
scanning ELECTRON
electron microscope
interventional RADIOLOGY

MAXILLARY RADIOGRAPHICALLY flying
radiographically flying europe

9. Causative Agent
9.1 bacteria

predominant ANAEROBES

growth mutans streptococci
mutans streptococci presence
mutans STREPTOCOCCI SALIVARY
mutans streptococci
staphylococcus aureus

conclude invasive GINGIVALIS
invasive gingivalis accelerates
gingivalis accelerates ATHEROSCLEROSIS
porphyromonas gingivalis

FUSOBACTERIUM peptostreptococcus
HELICOBACTER pylori
PNEUMOCOCCAL BACTEREMIA
actinomycetemcomitans rectus

9.2 behavioral factors

coffee SMOKER drinks
smoker drinks cranberry
SMOKELESS tobacco

various DIETARY sugars
dietary sugars
brushing flossing

10. Medication

10.1 immune system

MEDICATION allergies CONTRAINDICATIONS
ALLERGIES contraindications PRESCRIBED
allergies contraindications
EFFECTIVENESS PNEUMOCOCCAL vaccine
epinephrine anaphylaxis
PROINFLAMMATORY cytokines

10.2 antibiotics

contraindications prescribed amoxicillin
prescribed amoxicillin hydrocodone
AMOXICILLIN hydrocodone friend
amoxicillin hydrocodone
antimicrobial agents
tylenol codeine
broader spectrum

10.3 cardiac system

propofol PROPOFOL

10.4 anesthesia

valium triazolam
PARESTHESIAS solutions ARTICAINE
paresthesias solutions
viscous LIDOCAINE benadryl
XYLOCAINE CARBOCAINE

10.5 complementary and alternative medicine

complementary MEDICINE chiropractic
pharmaceutical herbal remedy

herbal remedy
HERBAL remedies
homeopathic REMEDY
vitamins NUTRITIONAL

10.6 cancer drugs

considers possible methotrexate
METHOTREXATE causative factor
methotrexate causative
intravenous BISPHOSPHONATES

10.7 other

NARCOTICS integral practicing
narcotics integral
documented PRESCRIBE carefully
SALINE sodium bicarbonate
saline irrigating
reflex anybody placebo
compounding pharmacy

11. Materials

11.1 bleaching

carbamide PEROXIDE
hydrogen peroxide
SODIUM bicarbonate
sodium perborate

11.2 resin materials

resins lining beverage
bonded RETAINERS scares
phosphoric PRIMER
scotchbond primer activated
scotchbond primer
primer activated primer
LESION geristore ionomer
geristore IONOMER location
geristore ionomer
ADHESIVE interface adhesive
adhesive flocked adhesive
expressed FLOCKED pooling
flocked pooling material
flocked adhesive expressed
flocked adhesive
adhesive expressed flocked
adhesive flocked
ULTRADENT continually syringes
continually syringes ultradent
continually syringes
oraseal ultradent continually
HELIOMOLAR filtek supreme
heliomolar filtek

11.3 microlux

expensive purchasing microlux
purchasing MICROLUX
purchasing microlux addent

addent microlux
microlux addent

11.4 anesthesia

anesthetic NEEDLES manufactures
needles manufactures manufactures
manufactures manufactures needles

11.5 mercury toxicity

reward MERCURY assured
contain mercury NEUROTOXIC

11.6 oral and maxillofacial surgery

FREEZE mineralized
FROZEN concentrate

11.7 other

blaylock fluoride
triangular shaped HANDPIECE
resorbable RESORBABLE
resilon percha
cotton pellet

12. Basic Sciences

12.1 inflammation

FUNCTION glucose homeostasis
glucose homeostasis
GLUCOSE homeostasis INFLAMMATION

12.2 anatomy

dentinal tubules
inferior ALVEOLAR distal
inferior alveolar
PERIPHERAL fibers
mental foramen
mucous membranes

12.3 other

molecular BACTERIAL antigens
breaking biofilm orifices
microbiology immunology
touted interproximal

13. Research

13.1 literature

pubmed indexed MEDLINE
PUBMED indexed
indexed medline
PERIODONTOL [*sic*] pubmed

13.2 methods and statistics

examiner reproducibility
RANDOMIZED controlled trials
RANDOMLY assigned
linear regression
confidence interval

inclusion criteria
causative factor coincidence
comparative EFFECTIVENESS
morbidity mortality
NUTRITION examination survey

13.3 funding

clinic federally FUNDED
federally funded
funded grants
principal investigator
council scientific affairs

Table. Keyword by category

Keyword (including variants and closely related words)	*Category*
1. plaque, plaques	1
2. atherosclerosis, atherosclerotic	1, 9
3. arterial	1
4. apolipoprotein	1
5. treadmill	1
6. cardiac, myocardial, endothelial	1
7. stenosis	1
8. pulses, pulsed	1, 4
9. episode, episodic	1
10. porphyria	1
11. alzheimer	1
12. pneumonia, pneumoniae, pneumococcal	1, 9, 10
13. chlamydia	1
14. shingles, zoster	1
15. resuscitate	1
16. syndrome	1
17. arthritis	1, 6
18. pulpitis, pulpal	2
19. maxilla, maxillary	2, 5, 8
20. obturation	2
21. medicine, medicate, medication	2, 10
22. exposure	2
23. fistula	2
24. surgically	2, 6
25. bleach	2
26. retainer, retainers	3, 11
27. orthodontics, orthodontists	3
28. mandible, mandibular	3, 5, 6
29. periodontally, periodontol [*sic*]	4, 13
30. calculus	4
31. pericoronitis	4
32. ultrasound	4
33. osteoclast, osteoclastic	4
34. coronal	5
35. nonrestorable	5

36. matrix	5
37. loosened	5
38. lateral, laterals, bilateral	5, 6
39. function, functions, parafunctional, dysfunction	1, 5, 12
40. temporary, temporarily	5
41. shades	5
42. carious, cariogenic	5
43. relines	5
44. splints, splinting	5
45. mineral, mineralized, demineralized, demineralization, remineralization	5
46. calcium	5
47. saliva, salivary	5, 6, 9
48. buffering	5
49. emergence	5
50. sequestrum	6
51. hematoma	6
52. osteomyelitis	6
53. bisphosphonate, bisphosphonates	6, 10
54. osteonecrosis	6
55. lesion	2, 6, 11
56. ulcers	6, 7
57. asymptomatic	6
58. invasive	6
59. tonsils	6
60. infect, disinfecting	5, 6
61. lichen	7
62. herpes	7
63. aphthous	7
64. electron	8
65. scanning	8
66. radiology, radiographically	8
67. anaerobes, anaerobic	6, 9
68. streptococci	9
69. gingivalis	9
70. fusobacterium, helicobacter, bacteremia	9, 12
71. smoker, smokeless	9
72. dietary	9
73. contraindications	10
74. prescribe, prescribed	10
75. effects, effectiveness	1, 10, 13
76. inflammation, proinflammatory	10, 12
77. amoxicillin	10
78. propofol	10
79. articaine, lidocaine, xylocaine, carbocaine	10
80. paresthesias	10
81. herbal	10
82. remedy	10
83. nutritional, nutrition	10, 13
84. methotrexate	10
85. narcotics	10
86. saline	10
87. peroxide	10

88.	sodium	11
89.	primer	11
90.	ionomer	11
91.	adhesive	11
92.	flocked	11
93.	ultradent	11
94.	heliomolar	11
95.	microlux	11
96.	needles	11
97.	mercury	11
98.	neurons, neurotoxic, neuropathy	1, 11
99.	freeze, frozen	11
100.	handpiece, handpieces	5, 11
101.	resorbable	11
102.	glucose	12
103.	alveolar	6, 12
104.	peripheral	1, 12
105.	medline	12
106.	randomized, randomly	13
107.	pubmed	13
108.	funded	13
